# ICSI in late matured oocytes, is it worth it? Study with laboratory, clinical and genetic evaluation results

**DOI:** 10.5935/1518-0557.20190091

**Published:** 2020

**Authors:** Laura T Vellez, Caroline Brogliato, Caroline Z Berton, Ivan H Yoshida, Caio P Barbosa, Emerson B Cordts

**Affiliations:** 1 Instituto Idéia Fértil de Saúde Reprodutiva, Santo André, SP, Brazil

**Keywords:** late matured oocytes, embryo development, blastocyst, genetic testing

## Abstract

**Objective:**

To compare laboratory results of embryo development from late matured oocytes in relation to mature oocytes in D+0.

**Methods:**

We carried out a cross-sectional study during the period from January to December 2018, in which we collected data through medical records analysis. 913 oocytes were collected and divided into 3 groups: group 1 - 643 MII oocytes; group 2 - 119 MI oocytes and; group 3 - 151 PI oocytes. These studied oocytes were from different maternal ages and infertility factors. The analyzed variables were fertilization rate, embryo cleavage, top quality embryos on the third day of development, blastocyst stage, top quality blastocysts, euploid blastocysts, top quality blastocysts and gestation. We documented the data, and performed the statistical analysis using the chi-square test (*p*<0.05).

**Results:**

All MII oocytes were injected (643); 103/119 MI oocytes and 88/151 PI oocytes that matured late in D + 1, were also injected. The fertilization rate of the three groups did not present statistical difference. The oocytes of group 1 had a statistically proven better prognosis than oocytes from groups 2 and 3 when compared, respectively, embryo cleavage (*p*=0.000), top quality embryos on the third day of development (*p*=0.000) and blastocyst formation rate (*p*=0.004). In the LMO group, there were no euploid embryos and, therefore, there no embryo transfer.

**Conclusion:**

Although late matured oocytes have made blastocyst formation possible, even if in low rates, there were no viable embryos for transfer.

## INTRODUCTION

The degree of oocyte maturity is an essential prerequisite for the success of fertilization and the viability of the embryo formed (Holubcová *et al*., 2019). Due to ovarian stimulation, it is common for a small percentage of oocytes collected - from 20% to 30% - to be immature; this is due to the stimulation of multiple follicles (Avrech *et al*., 1997).

Some immature oocytes, recovered in prophase I (PI) or metaphase I (MI) stages, have the potential to develop and become mature (Magli *et al*., 2006). Thus, *in vitro* nuclear maturation may be an alternative to increase the number of embryos obtained from the ovarian stimulation cycles, especially in cases of bad responders (Strassburger *et al*., 2004). Attempts have been made to promote the maturation of immature oocytes recovered from stimulated cycles and, despite successful fertilization, embryo development and pregnancy are still low.

The objective of the present study was to compare the embryo development and genetic analysis of blastocysts from late maturated oocytes (LMO) with the development of mature oocytes.

## MATERIAL AND METHODS

The study was carried out at Instituto Ideia Fertil de Saúde Reprodutiva, from January to December 2018. We collected data through the medical records analysis, obtaining 734 oocytes divided into three groups: group 1 - 643 MII oocytes; group 2 - 119 MI oocytes and; group 3 - 151 PI oocytes. The analyzed variables were fertilization rate, embryo cleavage, top quality embryos on the third day of development, blastocyst stage, top quality blastocysts, euploid blastocysts and gestation. 

Inclusion factors were: patients who underwent assisted human reproduction treatment in the period mentioned above, and who obtained, in addition to mature oocytes, late matured oocytes that underwent the intracytoplasmic sperm injection (ICSI) on the next day. 

Patients whom, upon oocyte retrieval yielded only mature oocytes and who did not underwent intracytoplasmic sperm injection (ICSI) were excluded from the study. The study did not raise data on maternal age and infertility factors. 

The data was documented and the statistical analysis was performed using the chi-square test (*p*<0.05). 

### Laboratory Reproductive Variables

The laboratory reproductive variables studied that correlated with late maturated oocytes were:

- Fertilization rate;
- Embryo cleavage;
- Top quality embryos on the third day of development;
- Blastocyst stage;
- Top quality blastocysts;
- Euploid blastocysts;
- Gestation.

## RESULTS

The embryo development and genetic analysis of blastocysts from LMO compared to mature oocytes on the day of oocyte retrieval were shown to have worse rates. All MII oocytes were injected (643); 103/119 MI oocytes and 88/151 PI oocytes that matured late in D+1, underwent ICSI. The fertilization rate of the three groups showed no difference. The variables that presented statistically significant values when compared to the three groups were: embryo cleavage (*p*=0.000) (MII 75%, MI 68% and PI 64%), top quality embryos on the third day of development (*p*=0.000) (MII 67%, MI 37.5% and PI 40%) and blastocyst formation (*p*=0.004) (MII 58%, MI 24% and PI 19%), respectively. Groups 2 and 3 did not present top quality blastocysts. 

The euploid embryo and pregnancy rate from group 1 was 47% and 54%, respectively. In LMO, groups there were no euploid embryos and, therefore, embryo transfer or pregnancy. When we compared group 1 with groups 2 and 3 as a single group, the results were the same. When comparing the groups of the LMO between them there was no statistical difference in all parameters analyzed. The LMO groups presented worse prognosis when compared to mature oocytes on the day of oocyte retrieval.

## DISCUSSION

We know the importance of the dream to generate a child in the couples lives and women who undergo assisted human reproduction. During this process, in addition to dealing with various issues and feelings, the day-to-day of embryo development monitored by these patients together with the laboratory, generates a big expectation and anxiety. In each oocyte collected and fertilized there is hope to obtain a quality embryo because of the dreamed gestation and a baby at home. 

In many cases, the number of mature oocytes (MII) collected and able to go through the fertilization process is not satisfactory. The patient, in some cases, such as poorly responding, may present immature oocytes in the prophase I (PI) or metaphase I (IM) stage during the oocyte retrieval, and it has been observed that there are immature oocytes which have the potential to develop and became mature *in vitro*. An alternative to increase the number of injected oocytes would be to perform ICSI in these LMO. This study aimed to compare the fertilization rate and embryo development of mature oocytes with LMO. 

The LMO are oocytes that have fertilization rates very similar to the mature oocytes, which could indicate a good embryo development and blastocysts formation. Our results are consistent with the literature (Chian *et al*., 2002), which shows that, despite successful fertilization, embryo development and pregnancy rates are still scarce.

The results obtained in the present study show that performing ICSI in LMO is not an effective alternative when the objective is to obtain euploid blastocysts and pregnancy. For both embryos from MI and PI, on the third day of embryo development, a percentage was obtained, which, although low, when compared to MII oocytes, can be considered good, having the oocyte quality as a parameter. Among these cleaved embryos, we found an even lower percentage of top quality embryos. Of all the cleaved embryos, few reached the blastocyst stage and none of them were top quality. 

We know that the most important thing to obtain gestation is not the quantity of embryos/blastocysts formed, but their quality (Oron *et al*., 2014). These blastocysts were submitted to genetic analysis and all presented aneuploidy results. Therefore, there was no embryo transfer from LMO. These results were the same when comparing mature oocytes with LMO as a single set: LMO from MI oocytes and those from PI oocytes showed no difference considering the analyzed variables. There are reports of gestations in patients with mature late oocytes, but they are very rare. For patients with only immature oocytes collected at the oocyte retrieval procedure or failure of fertilization, the technique of LMO injection could be indicated, but with the genetic analysis indication, to avoid psychological and emotional stress caused from a transfer with a negative result.

## CONCLUSION

The injection of LMO is a technique that enable immature oocytes to be viable for the ICSI procedure. The fertilization of these oocytes aims to obtain embryos for transference, and thus a gestation. However, our study showed the failure to form euploid blastocysts. We conclude that the efficacy of treatment with late matured oocytes is low, since embryo development is with poorer quality and aneuploidy blastocysts formation.
